# Combined Effect of Temperature and Oil and Salt Contents on the Variation of Dielectric Properties of a Tomato-Based Homogenate

**DOI:** 10.3390/foods10123124

**Published:** 2021-12-16

**Authors:** Andres Abea, Pere Gou, Maria Dolors Guardia, Sancho Bañon, Israel Muñoz

**Affiliations:** 1 IRTA-TA, Food Quality and Technology, Finca Camps i Armet, Monells, 17121 Girona, Spain; andres.abea@irta.cat (A.A.); pere.gou@irta.cat (P.G.); dolors.guardia@irta.cat (M.D.G.); 2 Department of Food Science and Technology and Nutrition, Faculty of Veterinary Science, University of Murcia, Campus Espinardo, 30071 Murcia, Spain; sanchoba@um.es

**Keywords:** tomato homogenate, dielectric properties, loss factor, dielectric constant, microwave, radiofrequency

## Abstract

Tomato-based processed foods are a key component of modern diets, usually combined with salt and olive oil in different ratios. For the design of radiofrequency (RF) and microwave (MW) heating processes of tomato-based products, it is of importance to know how the content of both ingredients will affect their dielectric properties. Three concentrations of olive oil and salt were studied in a tomato homogenate in triplicate. The dielectric properties were measured from 10 to 3000 MHz and from 10 to 90 °C. Interaction effects were studied using a general linear model. At RF frequencies, the dielectric constant decreased with increasing temperature in samples without added salt, but this tendency was reversed in samples with added salt. The addition of salt and oil increased the frequency at which this reversion occurred. At MW frequencies, the dielectric constant decreased with increasing temperature, salt, and oil content. The loss factor increased with increasing salt content and temperature, except in samples without added salt at 2450 MHz. Penetration depth decreased with increasing frequency and loss factor. Salt and oil contents have a significant effect on the dielectric properties of tomato homogenates and must be considered for the design of dielectric heating processes.

## 1. Introduction

Tomatoes and olive oil are two key components of the Mediterranean diet. Olive oil acts as an excipient food that increases the bioavailability of the nutrients present in tomatoes [1]. According to the European Commission, the production of processed tomatoes is expected to increase due to higher yields, whereas consumption will probably increase mainly due to growing demand for convenient and healthy food. EU exports of processed tomatoes are expected to increase 1% per year, with Spain, Italy, and Portugal contributing more than 90% of the production [2].

The thermal treatment of tomato-based products is usually carried out in heat exchangers; this operation produces characteristic cooked flavors while ensuring an extended shelf-life. However, the high temperatures at the surface of the tube and long processing times associated with conventional pasteurization are known to diminish the sensory and nutritional qualities of raw tomato products such as “salmorejo” or “gazpacho” [3]. Furthermore, tomato is a valuable source of vitamin C, which has been extensively reported to be a thermolabile nutrient, suffering important losses at high temperatures and over long processing times [4]. 

One of the most promising alternatives to conventional heating is dielectric heating. By subjecting the food matrix to an alternating electric field, volumetric heat is generated inside the food product. The volumetric heating overcomes the barrier of slow heat transfer rates and implies much faster processing, maintaining desirable food quality attributes such as nutrition and flavor [5].

The way materials interact with electric fields depends on their dielectric properties, which quantify a material’s ability to reflect, store, and transmit electromagnetic energy. Knowledge of these properties is essential in the design and implementation of dielectric heating processes, since they have a direct effect on the penetration depth of the electromagnetic waves in the food matrix and the heating rate [6].

In the presence of a dielectric, the intensity of an electric field is reduced by a factor of $\varepsilon_{r}$, which corresponds to the relative permittivity of the material and is expressed as a ratio with the permittivity of vacuum ($\varepsilon_{0}=8.854\times 10^{-12}J/V^{2}m$):

(1) $$\varepsilon_{r}=\frac{\varepsilon }{\varepsilon_{0}},$$

Multiple dielectric mechanisms contribute to the complex dielectric permittivity, which is defined as [7]:

(2) $$\varepsilon^{*}=\varepsilon^{\prime }-j\varepsilon^{\prime \prime },$$

The real component $\varepsilon^{\prime }$ is often referred to as dielectric constant; it is related to the capacity of a dielectric to polarize and orientate towards an applied electric field. The imaginary component $\varepsilon^{\prime \prime }$ is the loss factor, which is related to various energy dissipation mechanisms and thermal conversion. Thermal and dielectric properties are influenced by temperature, frequency of the alternating electric field, and food composition [7]. 

Recent developments in continuous-flow dielectric heating technologies have prompted a higher interest in the study of dielectric properties of fluid foodstuffs, including soy sauce [8], *salsa con queso* [9], mirin [10], vinegar [11], honey [12], fruit juices [13], chili sauce [5], and milk [14]. Fewer studies deal with the contribution of different ingredients to the dielectric properties of food products. Ahmed et al. [15] studied the influence of olive oil and other ingredients on the dielectric spectra of hummus. Franco et al. [16] studied the individual contributions of water, salt, and sugar to the dielectric properties of green coconut water. Luan et al. [17] studied the effect of oil, salt, sucrose, and bentonite on the dielectric properties of bentonite water pastes at MW frequencies. Regarding studies that have dealt specifically with tomato, De los Reyes et al. [18] measured the dielectric properties of fresh and osmotically dehydrated cherry tomatoes at 20 °C and 2450 MHz. More recently, Peng et al. [19] studied the effect of adding 0.2 g/100 g of NaCl and 0.055 g/100 g of CaCl_2_ on the dielectric properties of three different tomato tissues in the range of 300–3000 MHz over a wide range of temperatures. 

It is expected that a high-moisture product, such as a tomato homogenate, will have a lower dielectric constant with higher oil content and a loss factor that increases with salt and temperature. However, it is unclear if the effect of the emulsification of olive oil and the structural changes in the tomato tissue during cooking will affect this dielectric behavior at different frequencies. The objective of this work was to study the effect of temperature and different concentrations of salt and olive oil on the dielectric properties and penetration depth of a tomato homogenate at RF and MW frequencies.

## 2. Materials and Methods

### 2.1. Sample Preparation

Vine tomatoes were purchased from a local producer. After reception, tomatoes were washed with tap water through aspersion and processed using a cutter (model CUT-35; Castellvall, Girona, Spain), a 3 mm steel automatic sieve (model C80; Robotcoupe, Vincennes, France) and a colloid mill (model MZ-100; FrymaKoruma AG, Rheinfelden, Switzerland) working at 3000 rpm to obtain a homogeneous paste. Samples with three different concentrations of salt (0, 0.5, and 1%) and extra virgin olive oil (0, 5, and 10%) content were prepared and homogenized for 1 min using an immersion blender. Overall, 9 treatments were studied in triplicate (27 samples). These concentrations were chosen based on the common values found in commercial tomato-based products. Ingredients were combined to investigate possible interactions. Samples were left overnight at 4 °C for stabilization prior to analysis. 

### 2.2. Sample Characterization

To characterize the raw material, moisture content and total soluble solids of the tomato homogenate were measured in all samples. Moisture content was obtained by drying in an oven at 103 ± 2 °C until reaching constant weight [20]. Total soluble solids content was measured using a portable refractometer (model Quick-BrixTM 90; Mettler Toledo GmbH, Giessen, Germany) and expressed as °Brix. The tomato homogenate had a moisture content of 94.8 ± 0.8% and total soluble solids of 4.1 ± 0.1 °Brix. Particle size of the homogenized product was determined at room temperature using a laser diffraction particle size analyzer (model Mastersizer S; Malvern Instrument Ltd., Worcestershire, UK) with a measurement range of 0.01–3500 µm and an obscuration range of 8–15%. Particle size of the samples, expressed as Dv (90) (the size below which 90% of the sample lies), was found to be 836 ± 26 µm.

### 2.3. Measurement of Dielectric Properties

The measurement of dielectric properties (dielectric constant, $\varepsilon^{\prime }$, and loss factor, $\varepsilon^{\prime \prime }$) was carried out in all samples following the procedure described by Muñoz et al. [14]. Measurements were made using an open-ended coaxial line with a high-temperature probe, connected to a 5 Hz–3 GHz network analyzer (model E5061B, Keysight Technologies, Santa Rosa, CA, USA) through a DC-4 GHz electronic calibration module (model N7550A, Keysight Technologies, Santa Rosa, CA, USA), which was fan-cooled for higher thermal stability. The instrument was warmed up for 2 h and then calibrated with air, a short-circuit block (supplied by the manufacturer), and deionized water. Samples were contained in a stainless steel (316 L-s) 750 mL autoclave (106 mm in height and 108 mm in diameter), identical to the one described by Muñoz et al. [14] and pressurized to 500 kPa to avoid the formation of air bubbles and evaporation at high temperatures, which may affect the measurements. To ensure uniform temperature distribution, samples were heated slowly by adjusting manually the power of an electrical resistance. Temperature was monitored using a temperature probe (TESTO, Lenzkirch, Germany) placed near the probe. Dielectric measurements were taken every 10 °C from 10 to 100 °C, over a frequency range from 10 to 3000 MHz. Special attention was given to the dielectric properties at RF frequencies (27.12 and 40.68 MHz) and MW frequencies (915 and 2450 MHz) allocated for their use in industrial, scientific, and medical applications. All measurements were performed in triplicate.

### 2.4. Calculation of the Frequency at Which the Relationship between the Dielectric Constant and Temperature Reverses

For each sample, a linear regression of dielectric constant on temperature was fitted at each frequency, and the slopes were obtained (data not shown). The frequency at which the slope changed signs was taken as the reversion point $P_{r}$ of the sample.

### 2.5. Calculation of the Penetration Depth

Penetration depth is defined as the distance at which power decreases to 1/e of the initial value at the surface. It is a parameter that provides insight on the temperature uniformity during dielectric heating [8]. For each mixture of tomato homogenate, the penetration depth was calculated at 27.12, 40.68, 915, and 2450 MHz using Equation (3).

(3) $$d_{p}=\frac{c}{2\pi f\sqrt{2\varepsilon^{\prime }\left(\sqrt{1+{\left(\frac{\varepsilon^{\prime \prime }}{\varepsilon^{\prime }}\right)}^{2}}-1\right)}},$$

### 2.6. Statistical Analysis

Analysis of variance was performed for each selected frequency (27.12, 40.68, 915, and 2450 MHz) using the GLM procedure of the SAS statistical package (SAS Inst., Inc., Cary, NC, USA). The statistical linear model was:

(4) $$Y_{ijklm}=\mu +SC_{i}+OC_{j}+T_{k}+{\left(SC\times OC\right)}_{ij}+{\left(SC\times T\right)}_{ik}+{\left(OC\times T\right)}_{jk}+{\left(SC\times OC\times T\right)}_{ijk}+s_{ijl}+e_{ijklm},$$

where: $Y_{ijklm}$ is the observed value ($\varepsilon^{\prime }$, $\varepsilon^{\prime \prime }$, or $d_{p}$), μ is the overall mean, $SC_{i}$ is the salt effect at i concentration (i = 0, 0.5, and 1%), $OC_{i}$ is the oil effect at j concentration (j = 0, 5, and 10%), $T_{k}$ is the temperature effect at k °C (k = 10, 20, …, 100 °C), $s_{ijl}$ is the effect of the l sample within the group of samples with i salt concentration and j oil concentration, and $e_{ijklm}$ is the random residual of the model. 

The factor sample, nested in factor SC×OC, was used as the error term for testing the effects of SC, OC, and SC×OC. The residual was used as the error term for testing the effects of T, SC×T, and OC×T. For the reversion point, the statistical linear model was:

(5) $$Y_{ijk}=\mu +SC_{i}+OC_{j}+{\left(SC\times OC\right)}_{ij}+e_{ijk},$$

where: $Y_{ijkl}$ is the reversion point, μ is the overall mean, $SC_{i}$ is the salt effect at i concentration (i = 0, 0.5, and 1%), $OC_{j}$ is the oil effect at j concentration (j = 0, 5, and 10%), and $e_{ijk}$ is the random residual of the model. 

When a significant effect was encountered, least-squares means were compared using LSMEANS with Tukey’s test option. Differences were deemed significant at the 5% probability level. Values of least-squares means and standard deviation can be found in Tables S1–S13 of the Supplementary Materials.

## 3. Results and Discussion

### 3.1. Dielectric Constant ($\varepsilon^{\prime }$)

Values of $\varepsilon^{\prime }$ decreased with increasing frequency in all samples. This decrease is in accordance with the Debye relation [21]. The faster the alternation of the electric field, the more difficult it is for the water molecules to orientate towards it, which reduces the dielectric polarization and the energy storage [16]. This decline was sharper at RF frequencies, especially in samples with higher salt content at high temperatures, similarly to what was reported by Zhu et al. [13] in fruit juices. 

For tomato homogenate without salt or oil, the values of $\varepsilon^{\prime }$ ranged from 77.2–57.7 at 915 MHz and from 74.9–57.2 at 2450 MHz. These values are in accordance with previous results obtained by Peng et al. [19], who reported values ranging from 78 at 22 °C to 57 at 120 °C for pericarp, locular, and placental tissues of raw tomatoes at 915 MHz, and slightly lower values at 2450 MHz. 

The dielectric constant was significantly affected by salt and temperature at all frequencies (Table 1). There was a significant interaction between salt and temperature at RF frequencies. There was a significant effect from oil at all frequencies (except at 27.12 MHz) and from its interaction with temperature at MW frequencies.

**Figure 1:**
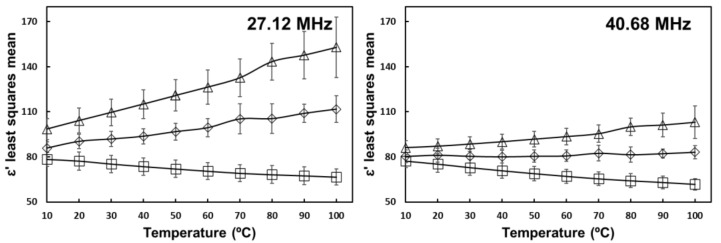
Salt and temperature interaction effect on the dielectric constant of tomato homogenate at 27.12 MHz and 40.68 MHz. (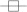) 0% salt (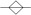) 0.5% salt (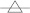) 1% salt. Error bars correspond to standard deviations.

Figure 1 shows the interaction effect of salt and temperature on $\varepsilon^{\prime }$ for the two evaluated frequencies in the RF region. At these frequencies, $\varepsilon^{\prime }$ increased with increasing salt concentration. Temperature did not significantly affect $\varepsilon^{\prime }$ in non-salted samples, but there was a significant increase in $\varepsilon^{\prime }$ with temperature in salted samples. Higher temperature and ion concentration increase ionic conductivity, which according to Zadeh et al. [22], causes an increase in dielectric dispersion, increasing the dielectric constant. 

**Figure 2:**
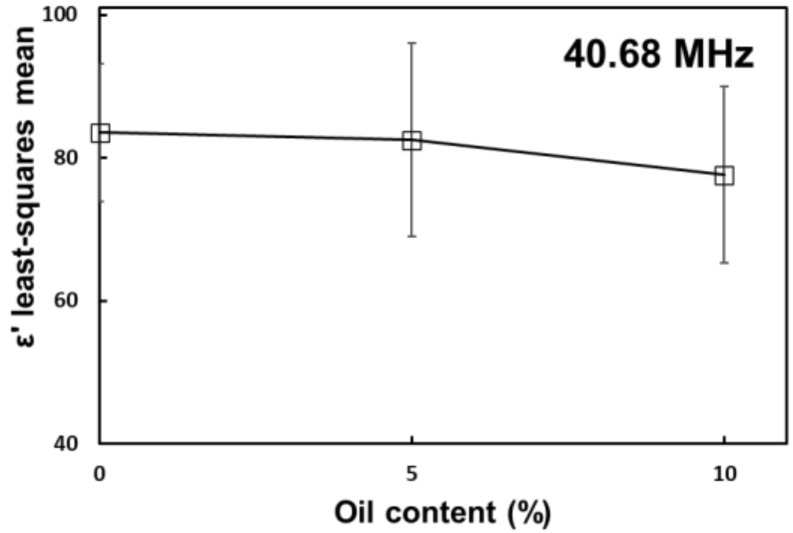
Main effect of oil content on the dielectric constant of tomato homogenate at 40.68 MHz. Error bars correspond to standard deviations.

At RF frequencies, there was a significant negative main effect from oil on the dielectric constant of tomato at 40.68 MHz (Figure 2). This effect was not significant at 27.12 MHz, possibly due to ionic conduction being the dominant polarization mechanism at low frequencies [23].

**Figure 3:**
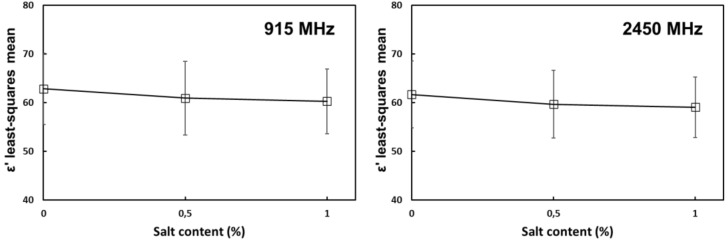
Main effect of salt content on the dielectric constant of tomato homogenate at 915 and 2450 MHz. Error bars correspond to standard deviations.

At MW frequencies, there was a significant negative main effect from salt on the dielectric constant (Figure 3). This shows that above a certain frequency between the RF and MW regions, the tendency was reversed and $\varepsilon^{\prime }$ started decreasing with increasing salt content. The negative effect from salt on the dielectric constant is caused by the binding of water molecules, which results in an obstruction of their polarization and overall energy storage [24].

**Figure 4:**
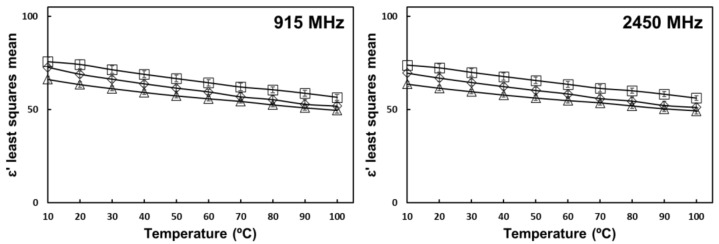
Oil and temperature interaction effect on the dielectric constant of tomato homogenate at 915 MHz and 2450 MHz. (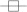) 0% oil (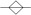) 5% oil (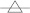) 10% oil. Error bars correspond to standard deviations.

The negative interaction effect of oil and temperature on the dielectric constant at MW frequencies is shown in Figure 4. The effect of oil is caused by water molecules being replaced by dielectrically inert molecules with almost no polarization [25]. The effect of temperature is caused by an increase in the Brownian motion of water molecules and a general reduction of viscosity in the mixtures, which in turn reduces the relaxation time and the energy storage [16].

The negative main effect of salt at MW frequencies was very small in comparison to that of oil. This is in accordance with what was reported by Peng et al. [19], where the authors did not find any significant differences in the dielectric constant of tomatoes after adding 0.2 g/100 g NaCl at 915 and 2450 MHz.

The opposite effect of temperature and salt content at RF and MW frequencies is similar to what was reported by Muñoz et al. [14] in milk, by Wang et al. [26] and Nelson and Bartley [27] in whey protein gels, by Luan et al. [17] in bentonite pastes, and by Guan et al. [28] in mashed potatoes. According to Nelson [29], the frequency at which the dielectric constant starts decreasing with increases in temperature or salt content marks the point from which dipole relaxation becomes the dominant loss mechanism over ionic conduction. 

**Figure 5:**
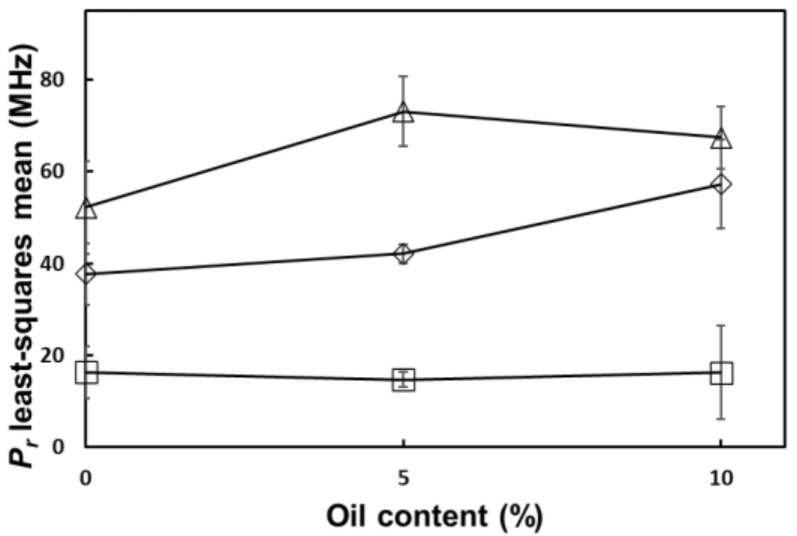
Salt and oil interaction effect on the reversion point of tomato homogenate (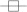) 0% salt (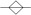) 0.5% salt (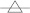) 1% salt. Error bars correspond to standard deviations.

Addition of ingredients changed the frequency at which this reversion occurred. Specifically, there was a statistically significant (*p* < 0.05) positive interaction effect from salt and oil on the reversion point (Figure 5). This effect is possibly caused by the increased loss in ionic conduction due to the addition of salt and the reduction in loss by dipole relaxation due to the addition of oil.

### 3.2. Loss Factor ($\varepsilon^{\prime \prime }$)

Values of loss factor showed a general tendency to decrease with increasing frequency and increase with increasing salt content and temperature. Values of $\varepsilon^{\prime \prime }$ of pure tomato homogenate ranged from 13.4 to 28.7 at 915 MHz and from 12.9 to 14.9 at 2450 MHz. This is in accordance with the values reported by Peng et al. [19], which ranged from 10 to 38 at 915 MHz and from 8 to 16 at 2450 MHz for pericarp, locular, and placental tissues of raw tomatoes.

Conversion of electromagnetic energy into heat occurs mainly due to dipole rotation and ionic conduction [7]:

(6) $$\varepsilon^{\prime \prime }=\varepsilon_{d}{}^{\prime \prime }+\varepsilon_{\sigma }{}^{\prime \prime },$$

where $\varepsilon_{d}{}^{\prime \prime }$ is the relative dipole loss and $\varepsilon_{\sigma }{}^{\prime \prime }$ is the relative ionic loss. The latter is directly proportional to the electrical conductivity (σ) and inversely proportional to the frequency (f):

(7) $$\varepsilon_{\sigma }{}^{\prime \prime }=\frac{\sigma }{2\pi f\varepsilon_{0}},$$

Ionic loss ($\varepsilon_{\sigma }{}^{\prime \prime }$) is the primary factor affecting dielectric loss at low frequencies. The relationship between $\varepsilon_{\sigma }{}^{\prime \prime }$ and f is characterized by a linear decreasing function in a log-log plot [30]:

(8) $$\log\varepsilon_{\sigma }{}^{\prime \prime }=a-b\log f,$$

where a and b are constants. The loss factor maintains this linearity as long as $\varepsilon_{\sigma }{}^{\prime \prime }$ is the primary loss mechanism, but it starts to deviate at high frequencies, where the frequency of the applied electric field begins to match the relaxation time of the water molecules, causing the dipole loss ($\varepsilon_{d}{}^{\prime \prime }$) to become more prevalent and increase until a maximum value at the point known as critical frequency [19,30]. 

**Figure 6:**
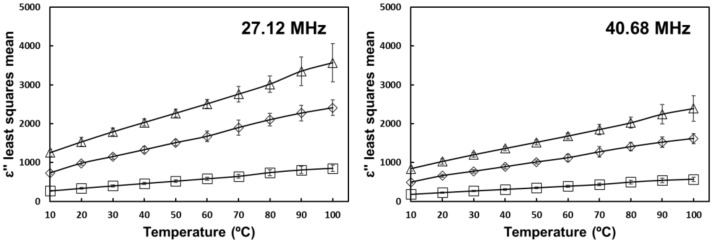
Temperature and salt interaction effect on the loss factor of tomato homogenate at 27.12 and 40.68 MHz. (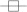) 0% salt (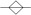) 0.5% salt (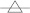) 1% salt. Error bars correspond to standard deviations.

**Figure 7:**
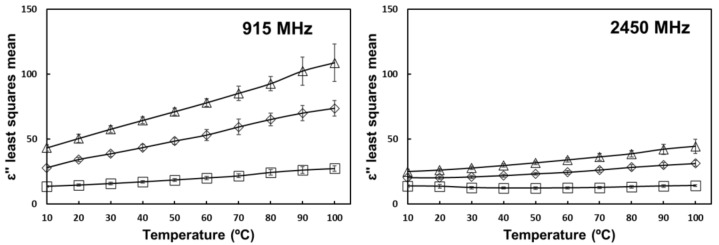
Temperature and salt interaction effect on the loss factor of tomato homogenate at 915 and 2450 MHz. (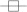) 0% salt (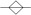) 0.5% salt (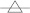) 1% salt. Error bars correspond to standard deviations.

The positive interaction effect of salt and temperature on the loss factor of tomato homogenate can be seen in Figure 6 for the studied frequencies in the RF region and in Figure 7 for the studied frequencies in the MW region. By adding salt, the increase in ion concentration increases the relative contribution of ionic loss [17]. At higher temperatures, the reduced viscosity increases ion mobility and overall ionic loss [14]. 

Loss factor did not significantly increase with temperature in samples without added salt at 2450 MHz. In this case, the high frequency and low salt content cause the dipole loss to become more prevalent. Dipole loss decreases with increasing temperature due to the relaxation times becoming increasingly shorter than the frequency of the applied electric field [19,22]. The opposing effects of temperature on dipole loss and ionic loss resulted in relatively constant values of $\varepsilon^{\prime \prime }$ across the studied temperature range.

In Figure 7, it can be seen that samples without added salt showed very little reduction in their values of $\varepsilon^{\prime \prime }$ from 915 MHz to 2450 MHz in comparison with those containing salt. For pure tomato homogenate at 10 °C, loss factor increased from 13.4 at 915 MHz to 14.9 at 2450 MHz. This is explained by the higher dipole contribution to the loss factor, which increased with increasing frequency in the studied range. The samples with added salt have a higher relative contribution of ionic loss; therefore, their loss factor values linearly decrease in the measured range according to equation 8 [17].

This dependence of ionic and dipole loss on temperature, frequency, and salt content is expected for high-moisture foods and is in accordance with what has been reported for many foodstuffs, including raw tomatoes [19], apples [31], green coconut water [16], milk [14], fruit juices [13], mirin [10], and soy sauce [8].

There was not a significant effect from oil on the loss factor at 27.12, 40.68, or 915 MHz. At 2450 MHz, there was a significant negative main effect; values decreased from 24.7 to 22.7 when increasing oil content from 0% to 10%. Samples with added oil had less polarizable molecules per unit volume and lower migration rate of ions due to the higher viscosity, which was expected to cause a general reduction of the loss factor. This reduction could only be appreciated at 2450 MHz, where the overall values of $\varepsilon^{\prime \prime }$ are small and the effect of dipole rotation becomes prevalent [15,24]. 

The results of this study are similar to the findings of Luan et al. [17] in bentonite pastes, where the authors postulate that $\varepsilon^{\prime }$ was mainly affected by oil content while $\varepsilon^{\prime \prime }$ was mainly affected by salt content. 

### 3.3. Penetration Depth ($d_{p}$)

**Figure 8:**
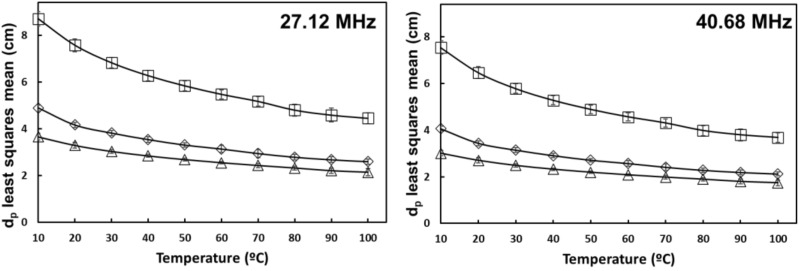
Temperature and salt interaction effect on the penetration depth of tomato homogenate at 27.12 and 40.68 MHz. (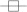) 0% salt (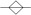) 0.5% salt (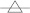) 1% salt. Error bars correspond to standard deviations.

**Figure 9:**
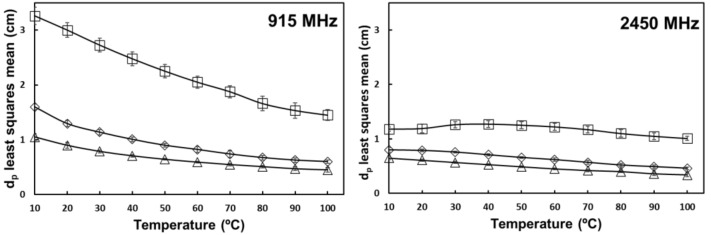
Temperature and salt interaction effect on the penetration depth of tomato homogenate at 915 and 2450 MHz. (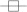) 0% salt (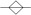) 0.5% salt (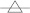) 1% salt. Error bars correspond to standard deviations.

Table 1 shows the level of significance of olive oil, salt, and temperature on the penetration depth of tomato homogenate at each selected frequency. Oil content had no significant effect on $d_{p}$. Values of $d_{p}$ decreased with increasing frequency, and there was a significant negative interaction effect from salt and temperature (Figure 8 and Figure 9). According to equation 3, $d_{p}$ is inversely dependent on the frequency and loss factor, so these results are in line with the expectations. Samples without added salt had an increasing penetration depth at 2450 MHz from 10 to 40 °C, in accordance with their increasing loss factor in that temperature range.

Similar results have been obtained for many foodstuffs, including mirin [10], soy sauce [8], inulin solutions [24], milk [14], and fruit juices [13]. Peng et al. [19] obtained values of $d_{p}$ ranging from 3.31 cm to 1.24 cm in tomato pericarp tissue at 915 MHz without added salt, and at 2450 MHz, increasing values from 1.24 cm to 1.45 cm at 60 °C and then decreasing again to 1.26 cm at 100 °C. Our results are in good agreement with these findings. 

According to Schiffmann [32], the thickness of a food product should not exceed 2 or 3 times its penetration depth to ensure heat generation inside the product. During continuous-flow dielectric heating, tomato homogenates with added salt would require lower tube diameters to maintain a uniform temperature rise, regardless of the oil content.

The increased penetration depth at lower frequencies (RF region) has the potential to reduce overheating, thus allowing for improved nutritional and sensory properties in vegetable homogenates. However, most of the research on continuous-flow dielectric heating has focused on MW frequencies. Coming years will probably see growing interest in exploring industrial applications in the RF region.

## 4. Conclusions

The dielectric properties of homogenates of tomato, salt, and olive oil were measured at four frequencies relevant to industrial dielectric heating applications. The dielectric constant was mainly affected by changes in temperature at all frequencies, by salt content in the RF range, and by oil content in the MW range. The loss factor was mainly affected by the salt content and temperature. The reversion point increased with the addition of salt and oil. The penetration depth generally decreased with increasing temperature, salt content, and frequency.

In dielectric heating processes, changes in dielectric properties resulting from new formulations must be taken into consideration because they will influence the temperature distribution and the heating rates associated with the thermal treatment. 

## Figures and Tables

**Table 1:** Significance level and root mean square error (RMSE) of each factor studied in the least-squares fitting of dielectric constant, loss factor, and penetration depth of tomato, olive oil, and salt mixtures.

Frequency (MHz)	RMSE	Salt	Oil	Temperature	Salt * Oil	Salt * Temperature	Oil * Temperature
		*ɛ*′					
27.12	5.09	***	NS	***	NS	***	NS
40.68	2.91	***	***	***	NS	***	NS
915	0.96	***	***	***	NS	NS	***
2450	0.94	***	***	***	NS	NS	***
		*ɛ*″					
27.12	127.86	***	NS	***	NS	***	NS
40.68	85.78	***	NS	***	NS	***	NS
915	3.77	***	NS	***	NS	***	NS
2450	1.34	***	***	***	NS	***	NS
					$d_{p}$		
27.12	0.13	***	NS	***	NS	***	NS
40.68	0.11	***	NS	***	NS	***	NS
915	0.06	***	NS	***	NS	***	NS
2450	0.03	***	NS	***	NS	***	NS
					$P_{r}$		
-	6.60	***	**	-	*	-	-

NS: Not significant ***: Significance at *p* < 0.001 level **: Significance at *p* < 0.01 level *: Significance at *p* < 0.05 level.

## Data Availability

Data is contained within the article or Supplementary Materials.

## Supplementary Materials

The following are available online at https://www.mdpi.com/article/10.3390/foods10123124/s1, Table S1: Least-squares mean value of dielectric constant at different combinations of temperature and salt content at 27.12 MHz. Lowercase and uppercase different letters indicate significant differences for temperature and salt variable, respectively (*p* < 0.05), Table S2: Least-squares mean value of dielectric constant at different combinations of temperature and salt content at 40.68 MHz. Lowercase and uppercase different letters indicate significant differences for temperature and salt variables, respectively (*p* < 0.05), Table S3: Least-squares mean value of dielectric constant at different combinations of temperature and oil content at 915 MHz. Lowercase and uppercase different letters indicate significant differences for temperature and oil variables, respectively (*p* < 0.05), Table S4: Least-squares mean value of dielectric constant at different combinations of temperature and oil content at 2450 MHz. Lowercase and uppercase different letters indicate significant differences for temperature and oil variables, respectively (*p* < 0.05), Table S5: Least-squares mean value of reversion point (MHz) at different combinations of temperature and oil content at 2450 MHz. Lowercase and uppercase different letters indicate significant differences for salt and oil variables, respectively (*p* < 0.05), Table S6: Least-squares mean value of loss factor at different combinations of temperature and salt content at 27.12 MHz. Lowercase and uppercase different letters indicate significant differences for temperature and salt variables, respectively (*p* < 0.05), Table S7: Least-squares mean value of loss factor at different combinations of temperature and salt content at 40.68 MHz. Lowercase and uppercase different letters indicate significant differences for temperature and salt variables, respectively (*p* < 0.05), Table S8: Least-squares mean value of loss factor at different combinations of temperature and salt content at 915 MHz. Lowercase and uppercase different letters indicate significant differences for temperature and salt variables, respectively (*p* < 0.05), Table S9: Least-squares mean value of loss factor at different combinations of temperature and salt content at 2450 MHz. Lowercase and uppercase different letters indicate significant differences for temperature and salt variables, respectively (*p* < 0.05), Table S10: Least-squares mean value of penetration depth at different combinations of temperature and salt content at 27.12 MHz. Lowercase and uppercase different letters indicate significant differences for temperature and salt variables, respectively (*p* < 0.05), Table S11: Least-squares mean value of penetration depth at different combinations of temperature and salt content at 40.68 MHz. Lowercase and uppercase different letters indicate significant differences for temperature and salt variables, respectively (*p* < 0.05), Table S12: Least-squares mean value of penetration depth at different combinations of temperature and salt content at 915 MHz. Lowercase and uppercase different letters indicate significant differences for temperature and salt variables, respectively (*p* < 0.05), Table S13: Least-squares mean value of penetration depth at different combinations of temperature and salt content at 2450 MHz. Lowercase and uppercase different letters indicate significant differences for temperature and salt variables, respectively (*p* < 0.05).
