# Supplementary material for: Combined Effect of Temperature and Oil and Salt Contents on the Variation of Dielectric Properties of a Tomato-Based Homogenate
Source: Foods. 2021 Dec 16;10(12):3124. doi: 10.3390/foods10123124 (PMC8701088; doi:10.3390/foods10123124)
Supplement: Supplementary file 1 [file foods-10-03124-s001.zip › Table S2.pdf]

Table S2. Least-squares mean value of dielectric constant at different combinations of temperature and salt content at 40.68 MHz. Lowercase and uppercase different letters indicate significant differences for temperature and salt variable, respectively ($P<0.05$).

Temperature (ºC)	Salt content (%)		
	0	0.5	1
10	$77.19\pm4.25^{A,a}$	80.4 ± 4.33^{AB}	$86.14\pm4.67^{B,abcd}$
20	$75.2\pm5.53^{A,ab}$	81.17 ± 4.65^{AB}	$87.10\pm4.97^{B,abcde}$
30	$72.89\pm5.20^{A,abc}$	80.5 ± 4.47^{B}	$88.53\pm4.93^{C,bcdef}$
40	$70.80\pm4.98^{A,abcd}$	80.11 ± 4.28^{B}	$90.06\pm4.96^{C,cdef}$
50	$68.89\pm4.85^{A,bcde}$	80.51 ± 4.00^{B}	$91.79\pm5.10^{C,def}$
60	$67.11\pm4.66^{A,cde}$	80.75 ± 3.86^{B}	$93.49\pm5.45^{C,efg}$
70	$65.47\pm4.46^{A,de}$	82.45 ± 5.26^{B}	$95.44\pm5.78^{C,fgh}$
80	$64.18\pm4.55^{A,de}$	81.52 ± 5.01^{B}	$99.96\pm5.78^{C,ghi}$
90	$63.05\pm4.23^{A,e}$	82.22 ± 2.89^{B}	$101.20\pm7.99^{C,hi}$
100	$61.81\pm3.79^{A,e}$	83.15 ± 4.32^{B}	$103.06\pm10.73^{C,i}$
