# Supplementary material for: Combined Effect of Temperature and Oil and Salt Contents on the Variation of Dielectric Properties of a Tomato-Based Homogenate
Source: Foods. 2021 Dec 16;10(12):3124. doi: 10.3390/foods10123124 (PMC8701088; doi:10.3390/foods10123124)
Supplement: Supplementary file 1 [file foods-10-03124-s001.zip › Table S4.pdf]

Table S4. Least-squares mean value of dielectric constant at different combinations of temperature and oil content at 2450 MHz. Lowercase and uppercase different letters indicate significant differences for temperature and oil variable, respectively ($P<0.05$).

Temperature	Oil content (%)		
(ºC)	0	5	10
10	73.79±2.44^{A,a}	69.59±1.76^{B,a}	63.73±1.31^{C,a}
20	72.32±2.21^{A,a}	66.93±1.89^{B,b}	61.53±1.36^{C,b}
30	69.93±2.01^{A,b}	64.56±1.76^{B,c}	59.65±1.19^{C,c}
40	67.72±1.86^{A,c}	62.36±1.72^{B,d}	57.85±1.21^{C,d}
50	65.63±1.74^{A,d}	60.28±1.63^{B,e}	56.21±1.35^{C,e}
60	63.57±1.64^{A,d}	58.42±1.53^{B,e}	54.82±1.34^{C,e}
70	61.41±1.95^{A,e}	55.93±2.03^{B,f}	53.65±1.24^{C,f}
80	60.14±1.27^{A,ef}	54.56±2.11^{B,f}	51.95±1.27^{C,f}
90	58.3±1.31^{A,f}	52.22±2.33^{B,g}	50.40±1.36^{B,g}
100	56.21±1.29^{A,g}	51.17±2.18^{B,g}	49.31±1.64^{B,g}
