# Supplementary material for: Combined Effect of Temperature and Oil and Salt Contents on the Variation of Dielectric Properties of a Tomato-Based Homogenate
Source: Foods. 2021 Dec 16;10(12):3124. doi: 10.3390/foods10123124 (PMC8701088; doi:10.3390/foods10123124)
Supplement: Supplementary file 1 [file foods-10-03124-s001.zip › Table S5.pdf]

Table S5. Least-squares mean value of reversion point (MHz) at different combinations of temperature and oil content at 2450 MHz. Lowercase and uppercase different letters indicate significant differences for salt and oil variable, respectively ($P<0.05$).

Salt content (%)	Oil content (%)		
	0	5	10
0	$16.33 \pm 5.71^{A,a}$	$14.74 \pm 1.60^{A,a}$	$16.22 \pm 3.00^{A,a}$
0.5	$37.71 \pm 6.63^{A,ab}$	$42.12 \pm 2.08^{A,b}$	$57.30 \pm 7.63^{B,b}$
1	$52.17 \pm 10.14^{A,b}$	$73.09 \pm 9.60^{AB,c}$	$67.41 \pm 6.84^{B,b}$
