# Supplementary material for: Combined Effect of Temperature and Oil and Salt Contents on the Variation of Dielectric Properties of a Tomato-Based Homogenate
Source: Foods. 2021 Dec 16;10(12):3124. doi: 10.3390/foods10123124 (PMC8701088; doi:10.3390/foods10123124)
Supplement: Supplementary file 1 [file foods-10-03124-s001.zip › Table S8.pdf]

Table S8. Least-squares mean value of loss factor at different combinations of temperature and salt content at 915 MHz. Lowercase and uppercase different letters indicate significant differences for temperature and salt variable, respectively (P<0.05).

Temperature (ºC)	Salt content (%)		
	0	0.5	1
10	13.74±0.63^{A,a}	28.05±1.10^{B,a}	43.44±2.46^{C,a}
20	14.68±0.79^{A,ab}	34.41±1.62^{B,ab}	50.69±3.22^{C,ab}
30	15.89±0.91^{A,ab}	38.82±2.07^{B,bc}	57.81±2.68^{C,bc}
40	17.21±1.05^{A,abc}	43.54±2.44^{B,cd}	64.54±2.64^{C,cd}
50	18.65±1.17^{A,abcd}	48.60±2.36^{B,de}	71.26±2.74^{C,de}
60	20.20±1.31^{A,abcde}	53.22±4.13^{B,ef}	78.11±2.94^{C,ef}
70	21.79±1.49^{A,bcde}	59.46±5.97^{B,fg}	85.37±5.62^{C,fg}
80	24.45±2.12^{A,cde}	65.23±4.88^{B,gh}	92.75±5.57^{C,g}
90	26.27±2.46^{A,de}	70.13±5.86^{B,hi}	102.48±10.82^{C,h}
100	27.43±2.22^{A,e}	73.84±5.93^{B,i}	108.82±14.42^{C,h}
